# The pupillary response discriminates between subjective and objective familiarity and novelty

**DOI:** 10.1111/psyp.12471

**Published:** 2015-07-14

**Authors:** Alexandros Kafkas, Daniela Montaldi

**Affiliations:** ^1^ School of Psychological SciencesUniversity of Manchester ManchesterUK

**Keywords:** Recognition memory, Pupil old/new effect, Pupil response, Novelty, Familiarity

## Abstract

The pupil response discriminates between old and new stimuli, with old stimuli characterized by larger pupil dilation patterns than new stimuli. We sought to explore the cause of the pupil old/new effect and discount the effect of targetness, effort, recollection retrieval, and complexity of the recognition decision. Two experiments are reported in which the pupil response and the eye fixation patterns were measured, while participants identified novel and familiar object stimuli, in two separate tasks, emphasizing either novelty or familiarity detection. In Experiment 1, familiarity and novelty decisions were taken using a rating scale, while in Experiment 2 a simpler yes/no decision was used. In both experiments, we found that detection of target familiar stimuli resulted in greater pupil dilation than the detection of target novel stimuli, while the duration of the first fixation discriminated between familiar and novel stimuli as early as within 320 ms after stimulus onset. Importantly, the pupil response distinguished between the objective (during an earlier temporal component) and the subjective (during a later temporal component) status of the stimulus for misses and false alarms. In the light of previous findings, we suggest that the pupil and fixation old/new effects reflect the distinct neural and cognitive mechanisms involved in the familiarity and novelty decisions. The findings also have important implications for the use of pupil dilation and eye movement patterns to explore explicit and implicit memory processes.

Discriminating old from new information is a critical human ability, as it guides our knowledge about the world and highlights the need to encode and process new information. This ability is central to performance on recognition memory tasks, in which participants are presented a set of old (previously studied) and new (unstudied) stimuli and are asked to decide whether each stimulus had been encountered during the previous study episode, or not. Decisions that a stimulus had been studied previously can be based either on recollection, which involves the retrieval of contextual details about the encounter with the stimulus (e.g., the place or time or other associated details) or on familiarity, which is the feeling of memory for a previously presented stimulus, without the retrieval of any contextual details (Mandler, 1980; Yonelinas, 2002). Familiarity can support accurate recognition without recollection, and it has been argued that familiarity‐based recognition decisions are supported by a neural system that produces distinct familiarity and novelty detection signals (for old and new stimuli, respectively; Kafkas & Montaldi, 2014). Temporally sensitive psychophysiological measures, such as pupil responses and eye movement patterns accompanying long‐term memory retrieval, may provide invaluable information further guiding our understanding of the cognitive processes involved in recognition memory decisions (Goldinger & Papesh, 2012; Kafkas & Montaldi, 2011, 2012; Otero, Weekes, & Hutton, 2011).

## Pupil Response in Recognition Memory: The Pupil Old/New Effect

Indeed, pupil dilation patterns can be an indicator of long‐term memory processing, both at encoding, by predicting the strength and the type of subsequent memory (Kafkas & Montaldi, 2011) and at retrieval, by discriminating between familiar and recollected stimuli (Kafkas & Montaldi, 2012). A pupil old/new effect has also been described (Vo et al., 2008), whereby the pupil response to old stimuli exceeds the response to those that are correctly rejected as new, in a recognition memory task. We have also reported (Kafkas & Montaldi, 2012) a pupil old/new effect discriminating between novel, familiar, and recollected stimuli at retrieval. In this study, a linear effect was found in which pupil dilation increased linearly from novel to familiar and recollected stimuli, with recollection producing the highest pupil dilation and novelty the lowest, with familiarity falling somewhere in between (for a similar finding, see Otero et al., 2011).

One important question relates to the source (or the cause) of the pupil old/new effect and its implications for familiarity and novelty detection, two processes vital for familiarity‐based recognition. All previous studies that have reported a pupillary old/new effect (e.g., Kafkas & Montaldi, 2012; Otero et al., 2011; Vo et al., 2008) have employed traditional recognition memory designs, in which participants are asked to discriminate old (studied) stimuli from new (unstudied) distractors. However, in a detection task like this, where participants are asked to discriminate a specific class of stimuli (e.g., old stimuli) from another (e.g., new stimuli), detection of a target stimulus may result in greater pupil dilation due to greater resource allocation and attention to the target (Granholm & Steinhauer, 2004; Kahneman & Beatty, 1966) or the greater rewarding value of target detection (Ariel & Castel, 2014; Kahneman & Peavler, 1969). This means that the pupil old/new effect may be created by the confounding effect of targetness resulting in greater pupil dilation for the old stimuli just because these are considered the target stimuli within a recognition memory task. Understanding the source of the pupil old/new effect is crucial if we are to be able to evaluate the usefulness of the pupil response as a measure of long‐term memory and its validity as a proxy for accurate (or inaccurate) recognition. We set out to explore this confound by designing a recognition memory task that discounts targetness and stresses both familiarity detection (i.e., detection of old stimuli) and novelty detection (i.e., detection of new stimuli).

A previous study (Kafkas & Montaldi, 2014) has been reported in which participants engaged in the identification of novel and familiar object stimuli in two separate tasks, emphasizing either novelty or familiarity detection. In this study, brain responses were measured using fMRI, while participants engaged in the evaluation of stimulus novelty or familiarity in two alternating task conditions. In the fMRI study (Kafkas & Montaldi, 2014), after an encoding session, participants were presented at retrieval in the familiarity task (FT) with 70% old (studied) target stimuli and 30% new foils, and they were asked to rate how familiar they found each stimulus, using a five‐point recognition memory scale with three increasing levels of familiarity strength (F1 = *weakly familiar*, F2 = *moderately familiar*, F3 = *strongly familiar*, N = *new*, R = *recollected*). In contrast, in the novelty task (NT), participants were presented with 70% new (unstudied) target stimuli and 30% old (studied at encoding) foils, and they were asked to evaluate how novel they found each stimulus, using a five‐point scale with three increasing levels of novelty strength (N1 = *weakly novel*, N2 = *moderately novel*, F3 = *strongly novel*, F = *familiar*, R = *recollected*). Responses in brain regions modulated by familiarity and novelty strength revealed two distinct pathways for familiarity and novelty detection, and showed that the signals are integrated in selective frontoparietal regions for the computation of the relative familiarity of a stimulus.

This design has the potential to disentangle two complementary components of familiarity‐based recognition memory—familiarity and novelty detection, which we propose contribute to a dual‐route recognition memory mechanism (Kafkas & Montaldi, 2014). It also treats both familiar and novel stimuli as targets in each task, emphasizing either the detection of familiarity or novelty, respectively. This eliminates the potential effect of targetness, as described above.

## Is the Pupil Old/New Effect Sensitive to the Objective or the Subjective Old/New Status of a Stimulus?

A further issue that the present experiments address relates to the capacity of the pupil response to discriminate between the objective and the subjective familiarity or novelty of a stimulus. We define objective familiarity or novelty as being derived from the veridical oldness or newness of a stimulus, irrespective of a participant's explicit old/new decision, while subjective familiarity or novelty is derived from the subjective old/new decision, irrespective of the veridical old/new status of the stimulus. The four recognition outcomes, according to signal detection terminology (hits, correct rejections, misses, and false alarms), describe a combination of objective and subjective old/new status of a stimulus. As noted in Table 1, in the case of hits and correct rejections (CRs), the objective and the subjective status of a stimulus are in agreement (e.g., the participants endorsed an old item as old), and therefore these are correct responses in the context of a recognition memory task. However, the incorrect responses, misses (M), and false alarms (FAs; bolded areas in Table 1) carry a disagreement^1^between the subjective old or new decision about a stimulus and its objective or true status. The question we seek to explore here is whether the pupil old/new effect is more sensitive to the objective or the subjective old/new status of the stimulus, or a combination of the two. Therefore, pupil responses to misses and false alarms (in both FT and NT conditions) can address this question because they contain a disparity between the subjective decision regarding the old/new status of the stimulus and the objective or true status. This would not be possible by simply examining hits and correct rejections, as in both cases the true (objective) status of old and new stimuli are consistent with the participants’ decision.

**Table 1:** Signal Detection Terminology

Task	Behavioral response	True status
FT		
Hits	Old	Old
Correct rejections (CR)	New	New
**False alarms (FA)**	**Old**	**New**
**Misses (M)**	**New**	**Old**
NT		
Hits	New	New
Correct rejections (CR)	Old	Old
**False alarms (FA)**	**New**	**Old**
**Misses (M)**	**Old**	**New**

*Note*. Areas in bold indicate recognition outcomes with a disagreement between the objective old/new status of a stimulus and the subjective response. FT = familiarity task; NT = novelty task.

The existence of brain regions sensitive to objective stimulus familiarity (independent of overt behavioral responses) has been investigated in a few fMRI and single‐cell recording studies. For example, Slotnick and Schacter (2004) identified two regions in the early visual cortex that were active for both hit and missed (i.e., forgotten) old stimuli. Within the medial temporal lobe (MTL), Daselaar, Fleck, Prince, and Cabeza (2006) reported activation in a posterior MTL region (including both the hippocampus and the parahippocampal cortex) related to the veridical oldness of a stimulus regardless of participants’ overt response. Moreover, a single‐neuron recording study (Messinger, Squire, Zola, & Albright, 2005) identified a population of neurons (54%) in monkey's inferior temporal cortex, which selectively responded to the presentation of original pairings independent of behavioral responses.

Eye movements have also been used as an index of memory, even in cases where conscious retrieval is absent (Hannula, Baym, Warren, & Cohen, 2012; Ryan, Althoff, Whitlow, & Cohen, 2000). In one study, for example, Ryan et al. (2000) showed that altered regions in repeated scenes attracted more fixations even when participants failed to overtly report these alterations. Previous studies have also attempted to use the pupillary response as an index of implicit recognition or mental processing with a particular focus on neuropsychological patients with selective deficits. Weiskrantz, Cowey, and Barbur (1999; see also Weiskrantz, 1998), for example, presented the case of a hemianopic patient with lesioned striate cortex (V1 and portions of V2) who demonstrated spared pupillary constrictions to isoluminant (equal luminance) stimuli, indicating residual visual functioning without awareness (as is the case with blindsight). In a further study, Lê, Raufaste, Roussel, Puel, and Démonet (2003) reported evidence of implicit face recognition in the pupillary response of a prosopagnosic patient, although an earlier study (Etcoff, Freeman, & Cave, 1991) had failed to show this finding.

More recently, Laeng et al. (2007) explored pupillary responses in three amnesic patients with damage including (but not isolated to) the hippocampus. They found pupillary old/new effects, despite participants’ poor performance on an explicit recognition memory task. Finally, two recent studies using a recognition memory paradigm (Montefinese, Ambrosini, Fairfield, & Mammarella, 2013; Otero et al., 2011) reported increased pupil dilation to false alarms (new stimuli falsely identified as old) relative to new stimuli (correct rejections) in healthy volunteers. Overall, these studies provide inconsistent results regarding the sensitivity of the pupil response to the subjective (e.g., false memory as indicated by a false alarm) or the objective status of old and new stimuli.

## The Present Study: Hypotheses

Here, we present two experiments, based on the design of the Kafkas and Montaldi (2014) study. Specifically, eye fixation patterns (Experiment 1) and pupil responses (Experiments 1 and 2) were measured, while participants engaged in the identification of novel and familiar object stimuli, in two separate tasks, emphasizing either novelty or familiarity detection. We hypothesized that if a pupillary old/new effect reflects the familiarity or novelty value of a stimulus, and not simply the stimulus targetness, then familiarity detection would result in greater pupil dilation than novelty detection. In Experiment 1, this was investigated using a rating task as used in Kafkas and Montaldi (2014), while in Experiment 2, where we wished to reduce decision complexity, the more standard yes/no paradigm was used in both familiarity (FT) and novelty (NT) conditions of the experiment. Given that research has shown that the pupillary response can also be modulated by task demands (e.g., Granholm, Asarnow, Sarkin, & Dykes, 1996; Karatekin, 2004; Porter, Troscianko, & Gilchrist, 2007), the comparison between Experiments 1 and 2 allows the investigation of whether the pupil old/new effect can be explained simply in terms of task demand. In Experiment 1, recollection responses to old stimuli were excluded from the analysis, as the main aim of the experiments reported here was to compare the pupil response to familiar versus novel stimuli, when the effect of the recollection component of recognition memory is eliminated (see Discussion). The comparison between pupil responses to recollected versus familiar stimuli is reported in a previous study (Kafkas & Montaldi, 2012). Finally, the sensitivity of the pupil response to both the objective (or veridical) and subjective old/new status of a stimulus is explored in the current research by comparing the pupil responses accompanying both misses and false alarms produced during the familiarity and novelty tasks.

## Method

### Participants

Two separate experiments were conducted using different samples. In Experiment 1, a total of 44 native English speakers gave informed consent and participated in the experiment in exchange for course credits. One participant was excluded from the analysis due to poor memory performance at retrieval, and six further participants were dropped due to incomplete pupil recordings (see below), leaving 37 participants (27 females) with a mean age of 20.3 years (*SD* = 1.97).

In Experiment 2, from the 34 participants who gave informed consent and participated in the experiment, four were excluded due to incomplete eye tracking recordings (excessive blinking or other artifacts) and one due to below chance performance in the novelty task. The remaining 29 participants (22 female) had a mean age of 20.1 (*SD* = 1.3). Both studies were approved by the School of Psychological Sciences Research Ethics Committee of the University of Manchester. No participant in the two experiments reported any systematic use of psychotropic medicines or drugs that would affect eye movements and pupil size.

### Stimulus Materials

The stimulus materials in both experiments consisted of 220 grayscale pictures (20 stimuli for practice) depicting natural and manmade objects, subtending a visual angle of 18.7^°^ horizontally and 14.05^°^ vertically, at presentation. Stimulus presentation was controlled through E‐prime (version 1.2) using a 15‐inch LCD Dell monitor running on 1,024 × 768 pixels resolution. Due to the sensitivity of pupillary responses to light intensity (Cheng, Rao, Cheng, & Lam, 2006), as well as to chromatic changes (Tsujimura, Wolffsohn, & Gilmartin, 2006), properties such as ambient light, stimulus brightness, contrast, as well as color of the presented visual items were controlled. As originally described in Kafkas and Montaldi (2011), this pool of stimuli consisted of pictures of single everyday items with uniform surfaces, presented in black and white in low contrast on a gray background (RGB = 130). Luminosity was matched using the luminosity tool in Adobe Photoshop (version 10.0.1) while also keeping a constant RGB level (red = 130, green = 130, blue = 130) across stimuli. Finally, levels of luminance emitted by each stimulus were recorded using a digital light meter (Meterman, LM631) placed in front of the computer screen. Any stimulus diverging by more than two standard deviations from the mean luminance level was discarded from the pool. Within each experiment, the stimuli were allocated randomly to each condition and were freshly randomized for each participant.

### Procedure and Design

**Figure 1:**
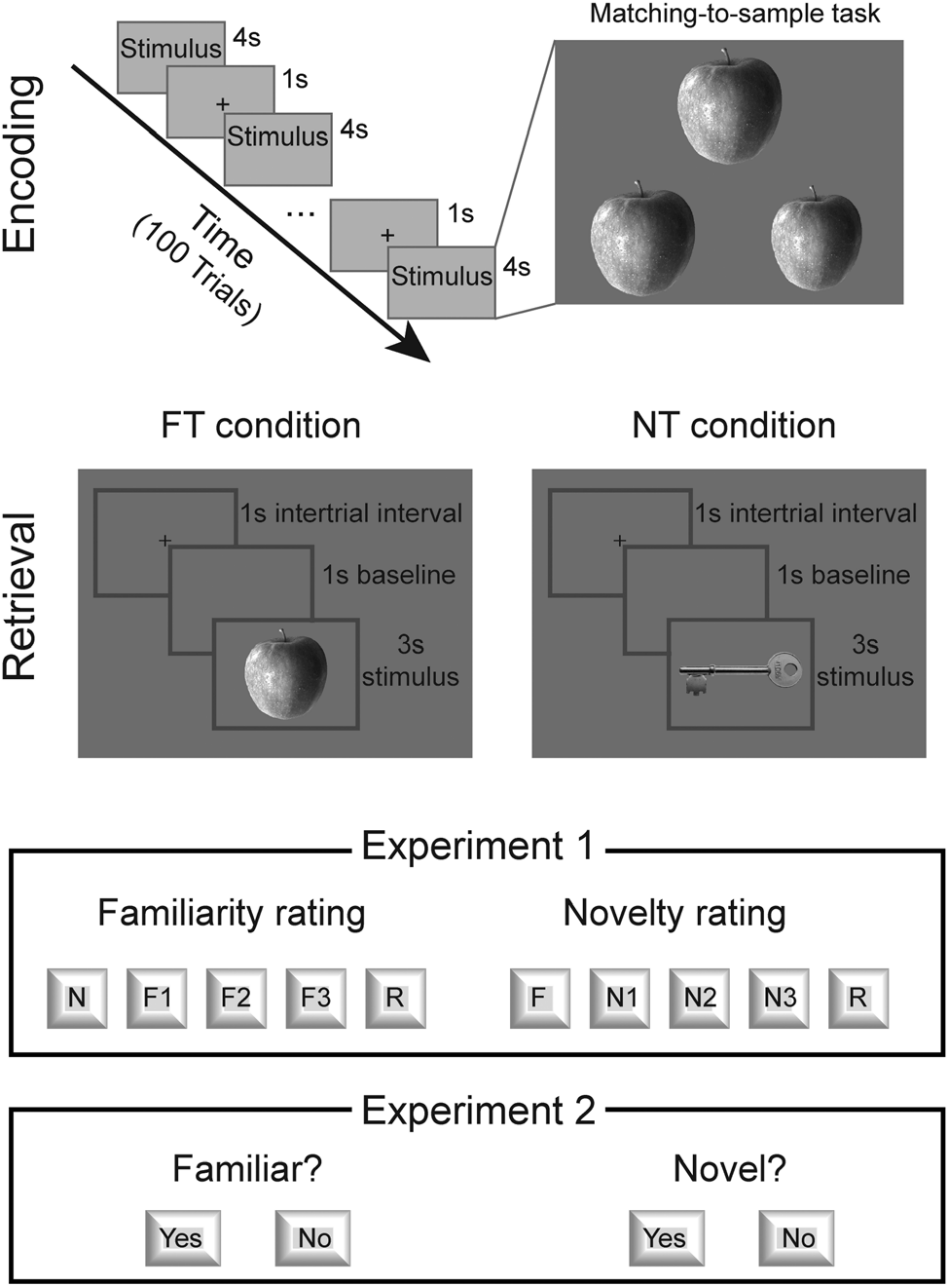
Design of Experiments 1 and 2. In both experiments, a perceptual matching‐to‐sample procedure was used at encoding. At retrieval, participants completed two tasks emphasizing either novelty (NT condition) or familiarity (FT condition) detection. In Experiment 1, familiarity and novelty decisions were taken using a rating scale while in Experiment 2, a simpler yes/no decision was used. Eye tracking data were recorded in both experiments during the FT and NT conditions.

In the two experiments reported here, we adapted a new recognition memory paradigm, described in a recent study (Kafkas & Montaldi, 2014) in which familiarity and novelty brain signals were discriminated using fMRI. The advantage of this design lies in its ability to measure novelty and familiarity detection in contexts/tasks that selectively emphasize novelty and familiarity processing, respectively. Experiments 1 and 2 follow almost identical designs and are therefore described together. The only difference between the two experiments is the number of the available responses at retrieval (see Retrieval below). In both experiments, participants completed an encoding and a retrieval phase within the same testing session. The design of the two experiments is illustrated in Figure 1.

#### Encoding

The encoding phase was identical in Experiments 1 and 2. Participants encoded a total of 100 object stimuli using a perceptual matching‐to‐sample task. In this task, each stimulus was presented as an image triplet depicting three copies of the same object, and for each triplet participants were asked to decide which of the two bottom images was identical to the top one (see Figure 1). In each trial, one of the bottom images differed very slightly either in size or in orientation with respect to the top image. The position of the matched target was randomly assigned for each trial. Participants were given 4 s per trial to indicate their response by pressing one of two buttons on a keyboard using both hands (1 for left, 0 for right). This encoding task is known to produce good memory performance, which relies more on familiarity memory and less on recollection due to the nature of the stimuli and the study instructions (see Kafkas & Montaldi, 2012, 2014; Montaldi, Spencer, Roberts, & Mayes, 2006). Encoding was followed by a 10‐min filled interval, during which participants engaged in a distractor task (containing an arithmetic and a verbal task).

#### Retrieval

After completing the encoding phase, participants were trained in the use of familiarity and novelty detection rating, and were also carefully trained to discriminate between instances of familiarity and recollection (see Kafkas & Montaldi, 2012; Migo, Mayes, & Montaldi, 2012). In both experiments, retrieval was carried out under two conditions: during a FT condition, emphasizing familiarity detection, and during a NT condition, emphasizing novelty detection. The only difference between the two experiments was the number of the available responses in the two conditions (i.e., decision complexity). In Experiment 1, a familiarity strength rating scale was used (F1: weak familiarity, F2: moderate familiarity, F3: strong familiarity) in the FT condition and a novelty strength rating scale (N1: weak novelty, N2: moderate novelty, N3: strong novelty) in the NT condition. Two further options, to report recollected stimuli (R) and correct rejections, were also available in both the FT and NT conditions (see Figure 1). Similarly, in Experiment 2, the conditions selectively emphasized either the detection of old stimuli (FT) or the detection of new stimuli (NT) but, in contrast to Experiment 1, a more simple yes/no decision was required (i.e., “Yes, it's familiar” in the FT condition and “Yes, it's novel” in the NT condition).

In both experiments, the order of the two retrieval tasks (FT and NT) was counterbalanced across participants, with half of the participants starting with the FT followed by the NT condition and half starting with the NT followed by the FT condition. Each of the two retrieval conditions (FT and NT) contained 70 target stimuli and 30 foils. Therefore, in the FT condition, 70 studied stimuli (from the encoding phase) were presented intermixed with 30 new foils, whereas in the NT condition, 70 new (unstudied) stimuli were presented intermixed with 30 studied stimuli. This ensured that equal numbers of target stimuli (familiar and novel) were presented in the two tasks. Each trial began with a gray screen (1 s), and the pupil data recorded during this period served as baseline pupil measures for the following trial. Following this, a fixation cross appeared for 1 s, followed then by the stimulus for 3 s, during which participants were instructed to provide a response. The allocation of stimuli to the two conditions (FT and NT) was freshly randomized for each participant.

In both experiments, participants were instructed to focus on item familiarity or item novelty decisions, without engaging in any kind of effortful recollection of the old items (i.e., an effortful memory search) but, critically, to report spontaneous recollections (in Experiment 1) by pressing the appropriate response button from the five available response options (Figure 1; for more details on this method, see Kafkas & Montaldi, 2012; Mayes, Montaldi, & Migo, 2007; Migo et al., 2012; Montaldi et al., 2006). If participants engaged in an unsuccessful search for recollective information, this may have affected the resulting pupil responses to familiar and novel stimuli. In general, the encoding procedure combined with these specific retrieval instructions ensures the generation of fewer recollections (see, e.g., Kafkas & Montaldi, 2012, 2014; Montaldi et al., 2006), and as the focus of the current experiments was on the comparison between familiarity and novelty, recollection responses were collected only to ensure that they did not confound other response categories, but are not analyzed (for a comparison of pupil responses and fixation patterns accompanying familiar and recollected stimuli, see Kafkas & Montaldi, 2012). In both Experiments 1 and 2, training (i.e., instructions and practice blocks) with each participant, before commencing the retrieval tasks, ensured a clear understanding of the procedure for familiarity and novelty decisions (rating in Experiment 1 and yes/no decisions in Experiment 2). Furthermore, to ensure the purity of the familiarity‐based recognition in Experiment 1, at training participants were asked to explain the rationale for their familiarity and recollection responses for each stimulus presented in the practice block, and corrective feedback was provided in the case of ambiguity.

#### Signal detection terminology

Signal detection terminology is used to describe participants’ behavioral responses in the two experiments. However, due to the differential emphasis of the two retrieval conditions (FT and NT) on old and new stimuli, hits, correct rejections, false alarms, and misses define behavioral responses to old and new stimuli differently in the FT and NT conditions. In general, a hit response indicates accurate detection of a target stimulus, whereas a correct rejection indicates accurate rejection of a foil item. Therefore, a hit response in the FT condition indicates accurate detection of an old stimulus (Hit_FT_), whereas a hit response in the NT condition (Hit_NT_), where the target stimuli are unstudied, indicates accurate detection of a new item. Accordingly, CRs in these experiments were correct rejections of new foils in the FT (CR_FT_), and of old foils in the NT condition (CR_NT_). Accordingly, false alarms, according to signal detection terminology, indicate misidentification of a stimulus as a target, whereas a miss denotes failure to detect a target. Therefore, false alarms (FA) were new stimuli in the FT condition (FA_FT_), but old stimuli in the NT condition (FA_NT_)—and in both cases were misidentified as target stimuli. Misses consisted of old stimuli deemed new in the FT condition (M_FT_), and new stimuli considered old in the NT condition (M_NT_). Table 1 summarizes this terminology for the two retrieval conditions (FT and NT) in the two experiments presented here. Finally, it should be noted that, as in any recognition memory experiment, the categorization of a stimulus as a hit, correct rejection, miss, or false alarm is carried out post hoc, based on the comparison between the actual status of a stimulus as old or new, and the behavioral response given by the participant. The participants did not receive any feedback with respect to the accuracy of their response at retrieval.

### Eye Tracking Recording and Analyses

#### Recording

Left eye pupillary responses and eye movements were recorded in both experiments using an ASL infrared eye tracking system (Applied Science Laboratories, Model Eye‐Trac 6000; sampling rate 60 Hz) during the two tasks (FT and NT). Before the beginning of the experimental session, a nine‐point standard calibration procedure was carried out with each participant. Furthermore, at the end of each session, the system was calibrated by placing a 4‐mm artificial (“sham”) pupil on the chinrest at the position of each participant's eye. This ensured the transformation of the pupil recordings into millimeters. Moderate ambient illuminance was kept constant, at about 250 lx, for each participant.

#### Preprocessing

Blinks and other losses in the raw eye signal were identified by the eye tracking software and were discarded from further analyses. Trials containing less than 60% of valid recordings were excluded (this happened for < 5% of the total number of trials in Experiment 1 and for < 4% of trials in Experiment 2), and participants with many excluded trials (i.e., more than 40% of the total number of trials) were removed from the analyses (six participants in Experiment 1 and four participants in Experiment 2). For the remaining subjects, pupil recordings that departed from the mean by more than three standard deviations of each trial were discarded, as these abrupt pupil dilation or constriction patterns are considered noise in the pupil signal (Beatty, 1982; Beatty & Lucero‐Wagoner, 2000; Kafkas & Montaldi, 2011). Overall, from the total number of the raw pupil recordings across participants, only 2.43% in Experiment 1 and 2.64% in Experiment 2 were discarded due to blinks or other artifacts. Due to the small proportion of discarded pupil traces, the pupil data were analyzed without applying any interpolation procedure; however, when linear interpolation was applied, as a check, identical results were obtained.

#### Measures

In both experiments, the peak pupil size for each trial was calculated as the average of three pupil recordings proceeding and three recordings following the maximum pupil value of the corresponding trial. The resulting peak pupil responses in each trial of the experiment were expressed as the deviation from the baseline pupil size, recorded during a period of 1,000 ms preceding each trial (Beatty & Lucero‐Wagoner, 2000). Baseline‐corrected pupil responses were also plotted across time for each response category, standardized to a length of 10 time points, from stimulus onset to the time of response. This standardization procedure was necessary as the timing of participants’ response varied from trial to trial. This ensured that the averaged time points for each response category across participants captured equivalent stages of cognitive processing, revealing pupil changes related to the decision being made and not to any postdecision confirmatory processing. Finally, from the raw eye movement signal, measures of spatial and temporal fixation properties were extracted. A spatial threshold of 1 degree of visual angle and a temporal threshold of 100 ms gaze time were used as the criteria by which fixations in the raw eye movement data were defined (Manor & Gordon, 2003). These include the number of fixations; the interfixation distance, which is the length of the saccadic movement between the fixation periods (in degrees of visual angle); and two temporal measures describing the time spent fixating (a) the duration of the first fixation, and (b) the mean gaze duration.

#### Analyses

In both experiments, recognition performance in the two retrieval conditions (FT and NT) was calculated by subtracting the proportion of false alarms from the corresponding proportion of hits (pHits − pFAs). Performance indices, response times (RT), peak pupil dilation, and fixation patterns were each analyzed in Experiment 1, using a two‐way repeated measures analysis of variance (ANOVA) with condition (FT and NT) and detection strength (three levels of familiarity and novelty) as the within‐subject factors. Similarly, performance, RT, peak pupil dilation, and fixation patterns in Experiment 2 were each analyzed by employing a paired *t* test contrasting the different response outcomes (hits, correct rejections, misses, and false alarms) in the two conditions. The pupil time series in the two retrieval conditions (FT and NT) were analyzed in both experiments for each response type (hits, correct rejections, misses, and false alarms) using two‐way ANOVAs with condition (FT and NT) and time (10 time bins) as the within‐subject factors. A standard significance level of *p* < .05 was adopted for all analyses and for the repeated measures ANOVAs the Greenhouse‐Geisser correction was used.

Finally, to further assess the temporal patterns of the pupillary responses across time and identify distinct temporal stages during the decision period, a principal component analysis (PCA) was applied to the pupil signal time‐course data (10 time bins) for the inaccurate responses separately (misses and false alarms). A varimax rotation method with Kaiser normalization was used and, as standard, factors with eigenvalues greater than 1 are reported (Kaiser's criterion; Kaiser, 1960) along with the percentage of the variance explained by each component. The PCA was applied only to misses and false alarms because, in the case of hits and correct rejections, the objective old/new stimulus status corresponds with the subjective experience, and therefore the pupil signal should reflect an undifferentiated summative response. In contrast, misses and false alarms indicate a disagreement between the objective old/new status of the stimulus and the explicit subjective experience. This makes these responses especially well placed for exploring the interaction between objective and subjective old/new information and its effect on the pupil signal.

## Results

### Behavioral Results

#### Performance

Participants accurately identified old and new stimuli in the FT and NT conditions, performing significantly better than chance in both conditions across both experiments (FT: *M* = .81 (*SD* = .07), *t*(36) = 27.1, *p* = .001 in Experiment 1 and *M* = .77 (*SD* = .06), *t*(28) = 12.94, *p* < .001 in Experiment 2; NT: *M* = .82 (*SD* = .11), *t*(36) = 20.62, *p* = .001 in Experiment 1 and *M* = .80 (*SD* = .11), *t*(28) = 16.38, *p* < .001 in Experiment 2). The proportions of correctly identified items for each condition (FT and NT), corrected for false alarms (pHits − pFAs), in the two experiments are presented in Table 2. As was expected, performance increased as participants reported increased levels of familiarity or novelty strength, *F*(1,36) = 98.31, *p* = .001, η^2^ = .73, in Experiment 1. Finally, participants performed equally well in both FT and NT conditions (Experiment 1: *F* < 1; Experiment 2: *t*(28) = 1.68, *p* = .11).

**Table 2:** Proportion of Trials, Performance, and Response Times

	Prop	H − FA	RT ms		Prop	H − FA	RT ms
Experiment 1							
FT				NT			
F1	.15 (0.09)	.04 (0.07)	1,727 (297)	N1	.17 (0.10)	.07 (0.07)	1,771 (322)
F2	.19 (0.09)	.11 (0.07)	1,664 (231)	N2	.26 (0.15)	.15 (0.11)	1,669 (351)
F3	.46 (0.18)	.31 (0.13)	1,295 (267)	N3	.49 (0.18)	.31 (0.12)	1,333 (271)
M	.11 (0.08)		1,515 (309)	M	.07 (0.06)		1,603 (419)
CR	.66 (0.14)		1,397 (228)	CR	.55 (0.25)		1,434 (315)
FA	.24 (0.07)		1,631 (350)	FA	.39 (0.11)		1,667 (309)

Experiment 2							
FT				NT			
Hits	.71(0.15)	.61(0.19)	976(185)		.83(0.11)	.54(0.21)	1,120(257)
FAs	.10(0.09)		1,057(350)		.29(0.18)		1,238(323)
CR	.89(0.10)		1,009(231)		.70(0.17)		1,140(257)
M	.28(0.15)		1,129(242)		.17(0.11)		1,327(335)

*Note*. Proportions were calculated based on the number of old items for hits and misses and on the number of new items for FA and CR in FT. In NT, proportions were calculated based on the number of new stimuli for hits and misses and on the number of old items for FAs and CRs. Numbers in parentheses are standard deviations. FT = familiarity task; NT = novelty task; prop = proportion of trials; H − FA = proportion of hits minus corresponding proportion of false alarms (i.e., Hit − FA); RT = response times.

#### RT

In Experiment 1, strong familiarity and novelty responses were characterized by shorter latencies than moderate or weak responses as revealed by the main effect of strength, *F*(1,36) = 86.78, *p* = .001, η^2^ = .71 (see Table 2). The main effect of condition was not significant (*F* < 1) illustrating that RTs for the hit responses in the two tasks (FT and NT) were matched. In Experiment 2, Hit_FT_ responses were faster than Hit_NT,_ *t*(28) = −3.73, *p* < .001. Additionally, CRs, FAs, and M were faster in FT than in NT (CR: *t*(28) = −2.97, *p* = .006; FA: *t*(25) = −2.87, *p* = .008; M: *t*(27) = −4.49, *p* < .001).

### Fixation Patterns

**Figure 2:**
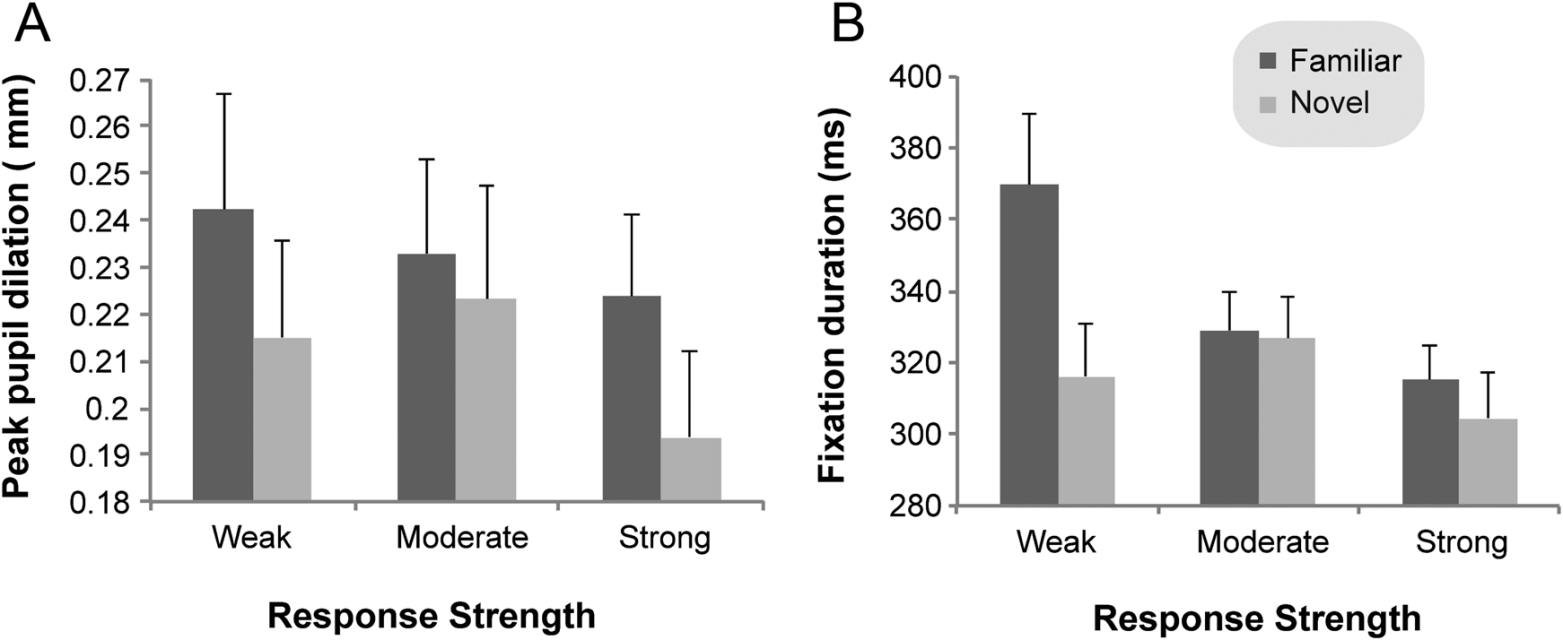
A: Peak pupil dilation (in mm) across the three levels of familiarity and novelty strength in Experiment 1. B: Duration of first fixation (in ms) across the three levels of familiarity and novelty strength in Experiment 1.

In Experiment 1, the first fixation was found to be significantly longer for the target familiar stimuli (in the FT condition) than for the target novel stimuli (in NT condition), *F*(2,72) = 4.38, *p* = .04, η^2^ = .11 (see Figure 2B). Importantly, this selective effect was evident as early as 320 ms following stimulus presentation. The number of fixations and fixation dispersion (i.e., interfixation distance), on the other hand, did not discriminate between familiar and novel stimuli in Experiment 1, but discriminated instead between responses of different strength, *F*(2,72) = 28.11, *p* < .001, η^2^ = .44 and *F*(2,72) = 5.85, *p =* .004, η^2^ = .14, respectively. Specifically, higher numbers of fixations characterized weakly and moderately familiar/novel stimuli compared to strongly familiar/novel ones (all *p*s < .001), while the interfixation distance data showed that strongly familiar and novel stimuli received less dispersed fixations than weakly (*p* = .004) or moderately (*p* = .003) familiar and novel stimuli.

### Pupil Measures

#### Experiment 1

**Figure 3:**
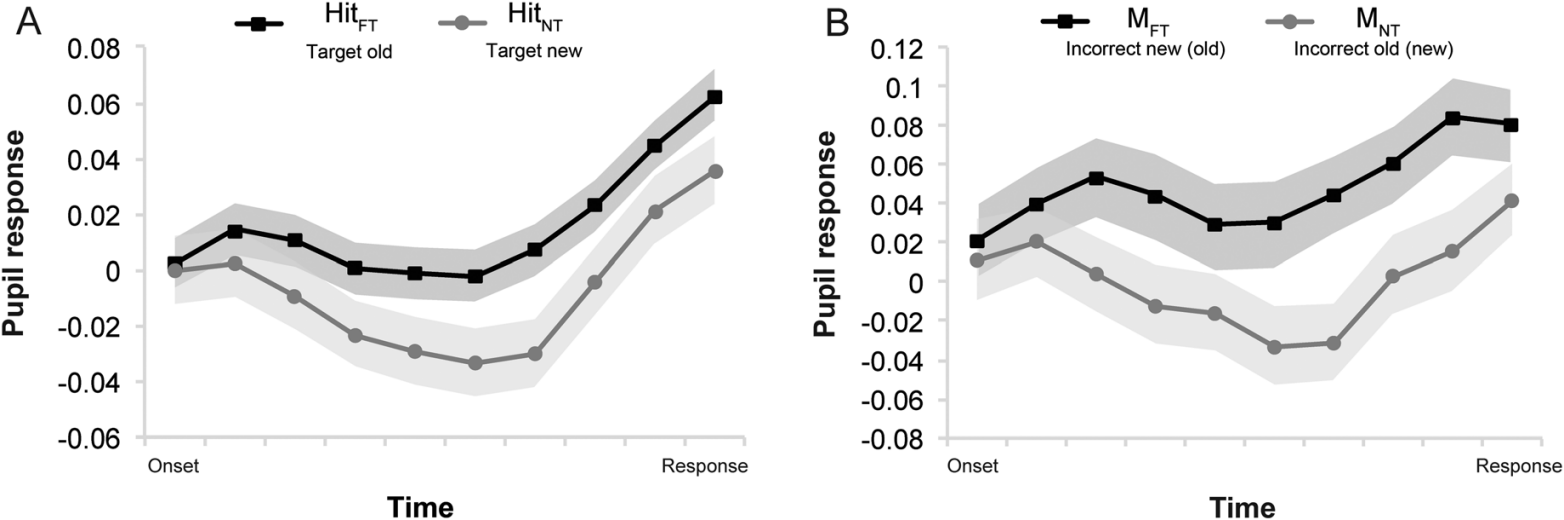
A: Pupil responses across time for target familiar (Hit_FT_) and target novel (Hit_NT_) stimuli in Experiment 1. B: Pupil responses across time for misses in FT and NT (M_FT_ and M_NT_, respectively) in Experiment 1. Shaded areas on the time series show standard errors of the mean.

Hits_FT_ produced larger peak pupil dilations than Hits_NT,_ *F*(1,36) = 4.39, *p* = 0.04, η^2^ = .11 (Figure 2A), while the strength of familiarity and novelty did not modulate the pupil effect, *F*(2,72) = 1.47, *p* = .24. Consistently, pupil dilation levels increased across time more for familiarity (Hit_FT_) than novelty detection (Hit_NT_; *F*(1,33) = 5.67, *p* = .02, η^2^ = .15) as was also indicated by a significant Condition × Time interaction, *F*(9,297) = 4.24, *p* = .008, η^2^ = .11 (see Figure 3A). Interestingly, M_FT_ (old items evaluated as new) produced larger pupil dilation across time than both M_NT_ (new items evaluated as old; *F*(1,28) = 4.51, *p* = .04, η^2^ = .14; Figure 3B) and Hit_NT_ (new items correctly classified as new; *F*(1,32) = 6.33, *p* = .017, η^2^ = .17). This suggests that the pupil response is particularly sensitive to the objective oldness of the stimulus, and discriminates familiar from novel stimuli even when the subjective experience does not discriminate between them.

#### Experiment 2

The comparison of the peak pupil dilation levels in Experiment 2 did not yield any significant result for any of the critical comparisons between the different response categories across the FT and NT conditions. Nevertheless, the time‐course analysis of the pupil response replicated and further refined the effects found in Experiment 1. The time course of pupil changes across the response period was compared between the two tasks for all response outcomes in a series of two‐way repeated measures ANOVAs with response type (response outcomes in FT and NT) and time (10 time bins) as the within‐subject factors.

**Figure 4:**
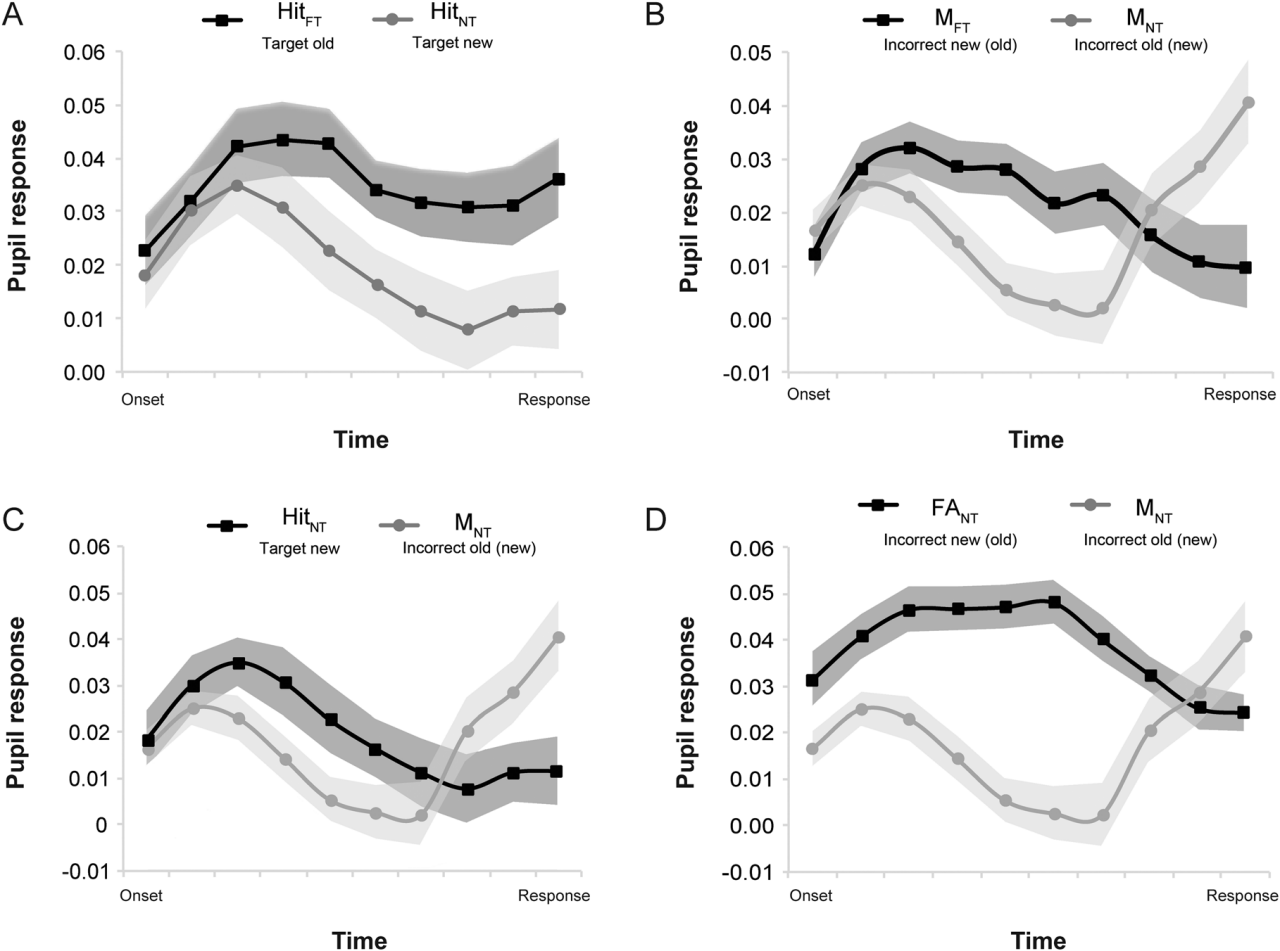
Comparison of pupil responses in Experiment 2 for different recognition outcomes in the FT and NT conditions. Hit_FT_ = hits in FT condition (target familiar stimuli); Hit_NT_ = hits in NT condition (target novel stimuli); M_FT_ = misses in FT condition (old stimuli deemed new); M_NT_ = misses in NT condition (new stimuli deemed old); FA_NT_ = false alarms in NT condition (old stimuli deemed new). Shaded areas on the time series show standard errors of the mean.

The comparison of Hits_FT_ (correctly identified old items) and Hits_NT_ (correctly identified new items) yielded a significant main effect of response, *F*(1,28) = 4.62, *p* = .04, η^2^ = .14, indicating elevated pupil responses for target old stimuli than target new ones (Figure 4Α). This finding was further supported by the significant Response Type × Time interaction, *F*(9,252) = 2.10, *p* = .03, η^2^ = .07, denoting differential pupil response patterns for old target stimuli and new target stimuli across the response period (as shown in Figure 4A). Post hoc *t* tests revealed that the differential pupil response between familiar and novel decisions became significant (all *p*s < .05) halfway through the response period (5th time bin) and remained distinct until the point of response. This pupil old/new effect was also evident within each condition, when comparing Hits_FT_ (i.e., old stimuli) with CR_FT_ (i.e., new stimuli; *F*(9,252) = 8.53, *p* < .001, η^2^ = .23) and Hits_NT_ (i.e., new stimuli) with CR_FT_ (i.e., old stimuli; *F*(9,252) = 4.11, *p* < .001, η^2^ = .13), showing consistently higher pupil dilation for old versus new stimuli. Overall, these findings replicate and extend the differential effects between familiar and novel stimuli found in Experiment 1.

The comparison of pupil responses for missed stimuli in the two tasks yielded a significant interaction between task response and time, *F*(9,252) = 3.14, *p* < .05, η^2^ = .10, indicating differential pupil changes across time for missed stimuli in the two tasks (M_FT_ and M_NT_). We used the PCA to further explore this effect. For both M_FT_ and M_NT_, two time‐linked factors (temporal components) were found that explain 92% of the variance of the pupil signal (Table 3). As shown in Figure 4B, M_FT_ (i.e., old stimuli deemed new) were characterized during the response period by greater pupil dilation levels than M_NT_ (new stimuli deemed old) until the 7th time bin. From the 8th time bin until the point of response (10th time bin), the reversed pattern is observed with pupil dilation reaching higher levels for new stimuli considered old (M_NT_) than old stimuli considered new (M_FT_).

**Table 3:** Factor Loadings (Eigenvalues) from the PCA of the Pupil Signal Across Time (10 Time Bins)

		Loadings across time bins									
	Variance	1	2	3	4	5	6	7	8	9	10
M_FT_											
Factor 1	69 (6.9)	**.95**	**.96**	**.97**	**.87**	**.80**	**.71**	.47	.31	.12	.01
Factor 2	23 (2.3)	.05	.11	.15	.35	.52	.61	**.83**	**.93**	**.97**	**.94**
M_NT_											
Factor 1	74.3 (7.4)	.08	.19	.36	.55	**.70**	**.79**	**.89**	**.95**	**.95**	**.89**
Factor 2	18.6 (1.86)	**.95**	**.95**	**.92**	**.79**	.65	.56	.37	.24	.17	.11
FA_NT_											
Factor 1	67.8 (6.8)	**.93**	**.95**	**.95**	**.85**	**.73**	.59	.46	.25	.09	.01
Factor 2	22.5 (2.25)	.05	.06	.20	.45	.58	**.73**	**.84**	**.92**	**.95**	**.94**

*Note*. Time bin loadings in bold indicate early and late components (factors). Variance = variance explained by each factor. M_FT_ = misses in FT condition (i.e., old stimuli reported as new); M_NT_ = misses in NT condition (i.e., new stimuli reported as old); FA_NT_ = false alarms in NT condition (i.e., old stimuli reported as new).

Finally, a comparison between Hits_NT_ (new stimuli) and M_NT_ (new stimuli deemed old) showed a differentiation of the pupil response starting from the 8th time bin, with larger pupil dilations characterizing missed, (i.e., deemed old), stimuli than new ones (Response × Time interaction: *F*(9,252) = 4.18, *p* = .017, η^2^ = .13; Figure 4C). The comparison between the pupil response for M_NT_ (new stimuli deemed old) and FA_NT_ (old stimuli deemed new) showed that FA_NT_ were characterized by larger pupil dilations than M_NT_, up until the 8th time bin, whereas for the 9th and 10th time bins the reverse pattern was observed (Response × Time interaction: *F*(9,252) = 2.43, *p* = .01, η^2^ = .08; Figure 4D). Indeed, the PCA carried out on the pupil response to FA_NT_ yielded a two‐factor solution explaining 91% of the variance of the pupil signal (Table 3), showing that the pupil responses up until the 5th time bin load on an earlier component (Factor 1), while the pupil responses during the remaining time bins (6th–10th) load on a later component (Factor 2). Overall, these findings refine the pupil old/new effect indicating that pupil dilation during the response period reflects both the objective (veridical) status of old/new stimuli (in the earlier component), and the subjective perception of the stimulus’ old/new status, closer to the point of the behavioral response reflecting that subjective decision.

## Discussion

In two experiments, we showed that familiarity and novelty decisions produced distinguishable pupillary dilation and fixation patterns. Furthermore, we showed that the pupil responses, produced while judging the novelty or familiarity of a stimulus, are sensitive to both the objective and the subjective old/new status of the stimulus, characterized by two separate temporal components. Specifically, in Experiments 1 and 2, familiarity decisions produced larger pupil dilations (both peak pupil dilations and relative changes across the response period) than novelty decisions. Moreover, the duration of the first fixation discriminated between familiar and novel stimuli as early as within 320 ms after stimulus onset, with familiar stimuli producing a longer first fixation even when familiarity was weak. Finally, as indicated by the consistency of the patterns of pupillary responses found across the two experiments, the differential pupil effects characterizing the response to familiar and novel stimuli reflects the underlying memory processing rather than the demands or complexity of the task employed (i.e., rating vs. yes/no decision).

A key aspect of the current experimental design is that familiarity and novelty decisions took place under two separate conditions, where the target stimuli were novel (NT) and where the target stimuli were familiar (FT). This means that the observed differences can only be attributed to the detection of the nature of the stimuli as familiar or novel and not to the detection of a stimulus as a target, as is the case in a traditional old/new recognition task, where only old items are normally the targets. Therefore these findings extend our understanding of the pupil old/new effect and highlight a potential role of the pupillary response as an online measure to explore both explicit and implicit memory phenomena.

### Source of the Pupil Old/New Effect

Previous research has shown that eye tracking measures (both pupil responses and eye movements) can be used as accurate indicators of long‐term memory, both at encoding and retrieval (Kafkas & Montaldi, 2011, 2012), and that they can discriminate between familiar, recollected, and new stimuli (Kafkas & Montaldi, 2012). Using word stimuli, Vo et al. (2008) have also found a pupil old/new effect at retrieval, with pupil dilations in response to old words being greater than pupil dilations in response to new words. This old/new effect was attributed by the authors to the greater episodic retrieval accompanying old responses, due to the need to recover specific contextual information associated with the old stimuli (i.e., recollection). However, our current findings do not support this proposal. In Experiment 1, the use of a more sensitive recognition test, which emphasized familiarity‐based recognition and carefully discriminated familiar from recollected stimuli, showed that even purely familiar stimuli were sufficient to elicit enhanced pupil dilation patterns—significantly larger than those elicited by new stimuli. Furthermore, Kafkas and Montaldi (2012) found a linear increase in the level of dilation at retrieval across new, familiar, and recollected stimuli, with the new stimuli eliciting the least pupil dilation and the recollected ones the greatest (for a similar finding, see Otero et al., 2011). Thus, although the retrieval of contextual information (recollection) may result in enhanced pupil dilation (as argued by Vo et al., 2008, and Kafkas & Montaldi, 2012), even familiarity‐based recognition alone, when recollection is absent, results in larger pupil dilation patterns relative to that produced by new items.

It is also important to note here that the observed effects cannot be explained by either the effort or the difficulty associated with the identification of familiar stimuli. First of all, participants performed equally well in both familiarity and novelty conditions in Experiments 1 and 2. In addition, RTs for familiar and novel hit responses in Experiment 1 were matched. However, in Experiment 2, participants were faster at identifying familiar than novel target stimuli. Assuming that RTs increase with more difficult decisions, the RT patterns in Experiment 2 would reflect greater difficulty or resource allocation in identifying new stimuli rather than old. According to the effort explanation (e.g., Granholm & Steinhauer, 2004), this should have produced greater pupil dilation for new relative to old stimuli, not the reverse as was found. To further ensure that the difference in RTs in Experiment 2 cannot account for the pupil old/new effect, we conducted the same comparison on pupil dilation patterns, excluding nine participants (*N* = 20) with notable differences in RTs between Hit_FT_ and Hit_NT_. This resulted in matched RTs between familiar and novel hits in the two conditions, *t*(19) = −1.58, *p* = .13, and despite this RT matching, the same pupil old/new effect was found, as was revealed in the main analysis of Experiment 2 (i.e., a greater increase in pupil dilation to familiar than novel stimuli, *F*(1,19) = 5.12, *p* = .03, η^2^ = 0.21). Therefore, the greater pupil dilation found here with familiar stimuli cannot be attributed to any potential differential difficulty associated with familiarity and novelty decisions.

If the cognitive effort hypothesis cannot provide a convincing explanation for the pupil old/new effect and the retrieval of associated contextual information cannot be the sole explanation of the phenomenon, what is the source (or the cause) of this effect? A possible explanation relates to the neural pathways supporting familiarity and novelty decisions. Using the same design as in Experiment 1, we have recently shown in an fMRI study (Kafkas & Montaldi, 2014) that familiarity and novelty signals are computed in two distinct sets of partially overlapping brain networks supporting the detection of familiar and novel stimuli. Thus, the contrasting pupil responses for old and new stimuli may reflect the adoption of different processing mechanisms for familiarity and novelty detection, which are supported by somewhat separate brain pathways.

We have recently suggested (Kafkas & Montaldi, 2014) that the organization of the memory system, which incorporates distinct familiarity and novelty signals, ensures that the detection and evaluation of novelty or familiarity is carried out by the brain in a way that both supports retrieval (of old information) and promotes further encoding (of new information). The pupil response may therefore reflect the combined output of encoding and retrieval, when familiar and novel stimuli are detected. Consistent with this, pupil dilation patterns at encoding have been found (Kafkas & Montaldi, 2011) to be greatly reduced for those stimuli that are better remembered at later retrieval (for a similar finding, see Naber, Frässle, Rutishauser, & Einhäuser, 2013). This clearly resembles the reduced pupil dilations accompanying the processing of new stimuli in the present and previous experiments (Kafkas & Montaldi, 2012; Otero et al., 2011; Vo et al., 2008).

One important aspect of the current design is that old stimuli in the FT condition were numerically dominant (relative to the new foils), whereas in the NT condition new stimuli were numerically dominant (relative to the old foils). This within‐condition imbalance ensured that the emphasis in each condition was maintained on either familiarity or novelty detection—the main conditions of interest contrasted in the analyses. As noted above, this design was also critical for the elimination of the potentially confounding effect of targetness when comparing the pupil responses. Although outside the scope of the present study, an interesting future question may explore to what extent the proportions of new and old stimuli and/or the instructions to focus only on familiar (in FT) or on novel stimuli (in NT) might influence the pupil response.

Interestingly, the duration of the first fixation also emerged as an early marker of discrimination between the processing of old and new information, as its duration was significantly longer for familiar stimuli than for novel ones. Moreover, this effect was evident even for the weakest category of familiarity, illustrating its considerable sensitivity to the new/old distinction. It has been argued that the existence of stored representations of a stimulus modulates visual scanning behavior early in the course of information processing (see also Ryan et al., 2007). Such a function must therefore require the rapid and efficient direct comparison between stored representations and incoming visual information. The current research strongly suggests that the output of the comparison is reflected in changes in fixation duration and pupil dilation (with increases in both for old information). Overall, the rapidity with which the difference in the duration of the first fixation becomes apparent highlights this as a very early marker of the accurate discrimination between old and new incoming information, suggesting the rapid triggering of distinct novelty and familiarity processing pathways. Taken together, the pupillometric and eye movement data support the idea that familiarity and novelty detection draw on distinct cognitive mechanisms that are differentially engaged very early on in stimulus processing.

### Two Temporally Distinct Pupil Response Signals Sensitive to the Objective and the Subjective Old/New Status of Incoming Information

Interestingly, the pupillary response data reported here not only reflected explicit recognition (Kafkas & Montaldi, 2012; Papesh, Goldinger, & Hout, 2012; Vo et al., 2008), but also emerged as a potentially important candidate measure for exploring unconscious (or implicit) memory. Specifically, the characteristic increase in pupil size found during the recognition of old stimuli was not only observable in the case of target familiar stimuli, but was also clearly evident in misses (objectively old/subjectively new) and FAs (subjectively old/objectively new) responses. This finding highlights the sensitivity of the pupil response to the implicit components of recognition memory, reflecting not only the subjective feelings of familiarity or novelty, but also the objective oldness or newness of a stimulus.

Interestingly, in Experiment 2, pupil response patterns revealed two temporally distinct processing stages related to recognition decisions (see Figures 4B,C,D). This was also confirmed in a PCA analysis, which showed that a two‐factor solution explains the majority of the variance of the pupil response accompanying misses and FAs in both conditions (FT and NT), with the exception of FA_FT_, which was characterized by a smaller number of trials. The PCA analysis confirms that the observed effects derive from sustained components within the pupil signal, and do not simply stem from the divergence of the pupil response at only two time points when the behavioral responses of interest are compared (e.g., the comparison between M_FT_ and M_NT_). The shape of the pupil response produced during the early recognition period appears to reflect the processing of the absolute oldness or newness of a stimulus. It is likely that during this period the veridical status of the stimulus (as old or new) and the availability or not of relevant representations is assessed. Following this relatively long period of more objective novelty and familiarity detection, a later, shorter processing component appears within the pupil time series. During this component, the subjective feeling of familiarity or novelty dominates and drives the pupil old/new effect so that stimuli inaccurately judged as old result in greater pupil dilation than those inaccurately categorized as new.

As discussed in the introduction, the utility of the pupil response as an indicator of implicit processing performance in neuropsychological patients (e.g., Laeng et al., 2007; Lê et al., 2003; Weiskrantz et al., 1999) has previously been explored. Furthermore, a “subjective pupil old/new” effect has been proposed (Montefinese et al., 2013; see also Otero et al., 2011) with the pupil dilation to FAs (subjectively old/objectively new) exceeding the dilation to CRs (objectively and subjectively new) in a standard recognition memory task. This literature is consistent with the short, late temporal component isolated in the pupil signal in the current study reflecting the subjective old decision. However, the current study is unique in illustrating a subjective pupil effect for new decisions (i.e., when old stimuli are falsely identified as new). Moreover, by exploring the time course of the pupil signal across the whole decision period, we further show for the first time that early on in processing the pupil response accurately reflects the objective status (new or old) of the stimulus, independent of the subjective decision that will shortly accompany these stimuli (for a similar effect in priming, see also Gomes, Montaldi, & Mayes, 2015). It is important to note that the pupil signal, while reflecting the objective status of a stimulus, is not itself accessible to the participant, and therefore does not directly influence the recognition response.

Collectively, the current findings support the existence of a mechanism sensitive to the objective status (old or new) of incoming information. As indicated by the findings reported here, the time course of pupil response across the decision period demonstrates that brain signals driven by this mechanism may inform autonomic reactions early in processing, reflected in pupil dilation patterns, discriminating old and new stimuli, irrespective of the later overt behavioral response. This mechanism, in turn, is likely to interact with a mechanism that is sensitive to the subjective perception of the status of the incoming information. Here, as the decision point approaches, pupil dilation reverts to reflect the subjective status of the incoming information.

### Conclusions

The findings reported here address a number of important issues relating to the use and interpretation of eye movement and pupil dilation data in the exploration of explicit and implicit memory systems. We show that familiarity and novelty detection and evaluation is characterized by distinct pupillary dilation and fixation patterns, which in the case of the latter offer a particularly rapid marker of the old/new status of incoming information. Critically, the design of the two experiments show that these effects cannot be explained in terms of task complexity, resource allocation, or the retrieval of associated contextual information (recollection). Instead, we suggest that, while these factors can influence pupil responses and fixation patterns under certain conditions, the pupillary and fixation old/new effects shown here are indicators of the engagement of distinct neural and cognitive mechanisms triggered by the detection and the evaluation of the status (new or old) of the incoming information. Finally, the pupil response is shown to be both a potentially useful indicator of implicit memory processing as well as a highly sensitive marker distinguishing between the early, objective and late, subjective processing of the old/new status of incoming information. Overall, this research illustrates the considerable opportunities that the measurement of pupil dilation and eye movement can offer to the study of memory systems.

## Footnote

1 The term *disagreement* is used here to stress the disparity between the true old/new status of a stimulus and the subjective decision. It does not relate to the experience of a participant at the time of making a recognition decision.
